# Supplementary material for: All-at-once RNA folding with 3D motif prediction framed by evolutionary information
Source: Nat Methods. 2025 Oct 3;22(10):2094–106. doi: 10.1038/s41592-025-02833-w (PMC12510878; doi:10.1038/s41592-025-02833-w)
Supplement: Supplementary file 3 — Source code to run CaCoFold-R3D. Data and results. [file 41592_2025_2833_MOESM3_ESM.gz › supplemental_material/results/CaCoFold-R3D/CaCoFold-R3D_Rfam/RF03013_nadA.cacofold.R2R.sto.pdf]

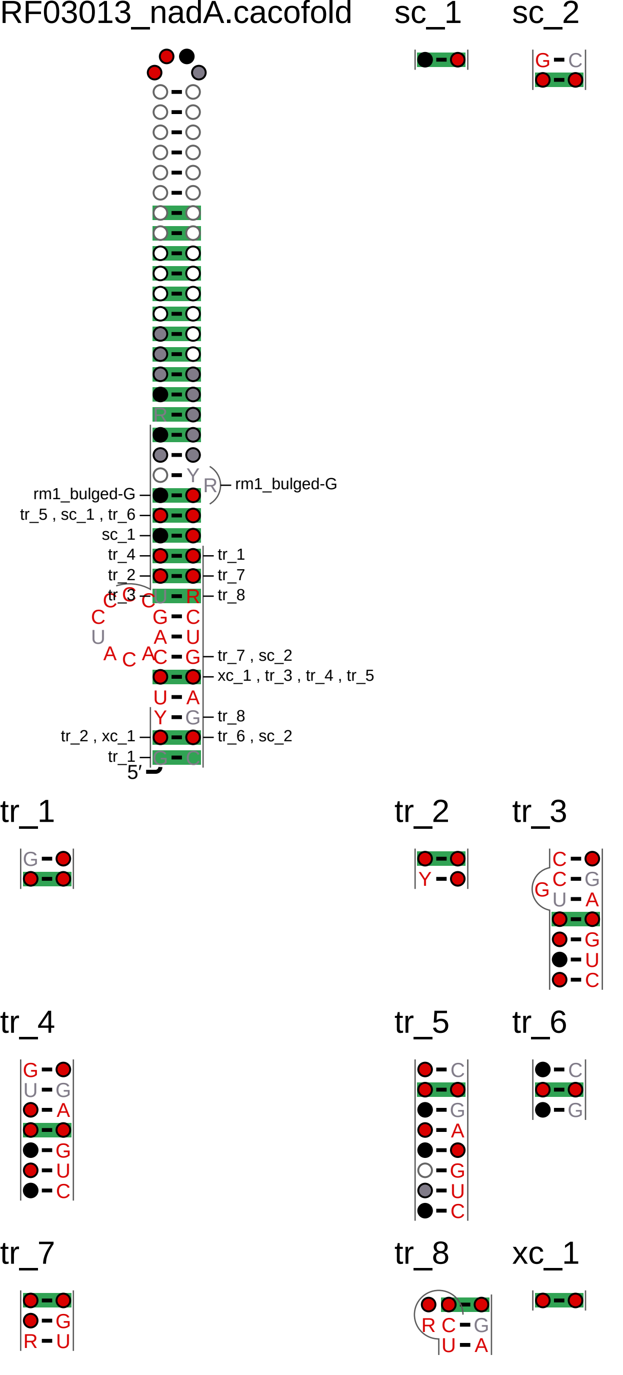

RF03013_nadA.cacofold
sc_1
sc_2
rm1_bulged-G
tr_5 , sc_1 , tr_6
sc_1
tr_4
tr_2
tr_3
tr_1
tr_7
tr_8
tr_7 , sc_2
xc_1 , tr_3 , tr_4 , tr_5
tr_8
tr_2 , xc_1
tr_6 , sc_2
tr_1
5′
tr_1
tr_2
tr_3
tr_4
tr_5
tr_6
tr_7
tr_8
xc_1
